# The epidemiology of hip and groin pain and Femoroacetabular Impingement Syndrome (FAIS) in male and female Gaelic games players

**DOI:** 10.1371/journal.pone.0309027

**Published:** 2024-09-25

**Authors:** Shauna Jordan, Clare Lodge, Ulrik McCarthy-Persson, Helen French, Catherine Blake

**Affiliations:** 1 School of Public Health, Physiotherapy and Sports Science, University College Dublin, Dublin, Ireland; 2 Department of Health and Sport Sciences, South East Technological University, Carlow, Ireland; 3 School of Physiotherapy, Royal College of Surgeons in Ireland, Dublin, Ireland; Iuliu Hațieganu University of Medicine and Pharmacy: Universitatea de Medicina si Farmacie Iuliu Hatieganu, ROMANIA

## Abstract

**Objectives:**

Hip and groin pain is common in Gaelic games players, but data are limited to elite males with poor representation of females. The aim of this study was to examine the prevalence, severity and factors associated with hip and groin pain and Femoroacetabular Impingement Syndrome (FAIS) in male and female Gaelic games players.

**Methods:**

A representative national sample of Gaelic games players completed a survey providing demographic information and details related to self-reported episodes of hip and groin pain and FAIS diagnosis within the last year. Players from multiple age grades, codes (Football/Hurling/Camogie) and levels of Gaelic games were included.

**Results:**

A total of 775 players responded to the survey. The annual prevalence of hip and groin pain was 54.8%. Almost half of players (48.8%) continued to participate in sport, while 18.7% ceased participation and 32.5% reported reduced participation. Although 40% of episodes lasted no longer than 3 weeks, there was a high recurrence rate (33.5%). FAIS was reported by eight players, representing 1.9% of hip and groin complaints. Logistic regression models indicate male sex, playing both codes of Gaelic games and participating in additional sport were significant factors in predicting hip and groin pain.

**Conclusion:**

Hip and groin pain is prevalent in Gaelic Games with FAIS accounting for a small proportion of cases. However, consideration of indicators of severity (participation impact/symptom duration/medical attention) is essential in understanding the context and magnitude of these hip and groin issues. Male players and players engaging in multiple sports are more likely to experience hip and groin pain.

## Introduction

Hurling/Camogie and football are codes of Gaelic games, Ireland’s national sport, engaged by over a half a million registered players worldwide. Despite rising international participation and recognition, with many players training to elite levels, Gaelic games remain an amateur sport. Hurling, played by males, and camogie, the female equivalent, are played with a stick, called a hurley, and a ball called a sliotar, similar in size to a hockey ball, while Men’s and Ladies Gaelic football is played with a round football, slightly smaller than a soccer ball. In all codes of Gaelic games, players play at local club level with some also playing for their school/college and a small proportion are selected to play at elite county level. There are also multiple age grades from underage to adult, with players often playing in more than one grade, level and code [1–3].

Hip and groin pain is common in sports involving explosive movements, change of direction and kicking [4–6] but consensus on the extent of hip and groin problems in athletes is hampered by heterogeneity in injury definitions, terminology and outcomes. There is little epidemiological data published on Gaelic games particularly at a broad population level that would represent most players. Hip and groin pain in Gaelic games has only been reported as a proportion of total injuries in general injury surveillance studies and these studies are limited to elite males. Time-loss groin/pelvic injury range from 6.3% to 14.4% in hurling and Gaelic football [7–10], while hip injury ranges from 2.3% to 7.7% [7, 9, 10]. Although female codes are growing in representation, only one study reported hip and groin prevalence in Ladies football, where 5% of injuries were related to the hip and groin region [11]. Overall, there is a dearth of information pertaining to prevalence of hip and groin pain in female Gaelic games players and a limited account of the wider population of male players, particularly at a local club level.

Time-loss and requirement for medical attention are criteria commonly used in epidemiological studies to define injury. Time-loss represents the period of absence from the date a player is forced to withdraw from their sport until the date of return to play and may underestimate the prevalence of a condition or injury. Prevalence rates as high as 38% to 59% have been reported for hip and groin pain in fields sports when non time loss injuries are included [12–14]. The International Olympic Committee (IOC) consensus statement on recording and reporting epidemiological data acknowledged that broader injury definitions are needed to capture all individuals with a particular health complaint, accounting for players with ongoing pain and symptoms who continue to participate in sport, potentially with reduced performance or participation. Furthermore, time-loss and requirement for medical attention also serve as indicators of severity but it is recommended that self-reported symptoms and consequences of injury are included when assessing severity to understand the extent of the problem [15].

Among many proposed intrinsic and extrinsic factors, the demographic profile of an athlete with hip and groin pain suggests male sex, increasing age and higher level of play are risk factors for developing hip and groin pain [4, 16, 17].This has been reported extensively in soccer players, but the contextual factors associated with Gaelic games players with hip and groin pain are unknown.

Femoroacetabular Impingement Syndrome (FAIS) is characterised by the presence of symptoms, clinical signs and relevant imaging findings [18]. Many studies report on the frequency of radiographic findings associated with cam morphology in athletes, but few studies have investigated the prevalence of the clinical FAIS disorder. Although the diagnostic criteria were not stated, FAIS diagnosis represented from 0.7% to 1.4% of cases of hip and groin pain across a multitude of sports in the National Collegiate Athletic Association (NCAA) in the United States [19–21]. While an exponential rise in uptake of surgery for FAIS has been noted in athletic populations worldwide in the last decade [18], the prevalence of the disorder is unknown in Gaelic Games.

The aim of this study was to investigate the prevalence, severity and factors associated with hip and groin pain and FAIS, self-reported in a representative sample of male and female Gaelic games players. Understanding the extent of any injury problem represents the first step in the sequence of prevention [22],therefore, the provision of this epidemiological data is a critical step to guide future research on hip and groin pain in Gaelic games and inform the development of preventative strategies.

## Methods

### Design

This cross-sectional epidemiological study involved surveying a national sample of Gaelic games players at the end of the 2019 season (September/October) and beginning of the 2020 season (January -March). The Strengthening of Reporting Observational Studies in Epidemiology for Sport Injury and Illness Surveillance (STROBE-SIIS) guidelines were utilised to report the findings of this study [15]. Ethical Approval for this study was obtained from The Human Research Ethics Committee in University College Dublin (Ref no: LS-19-13-Jordan-Blak).

### Participants

**Fig 1:**
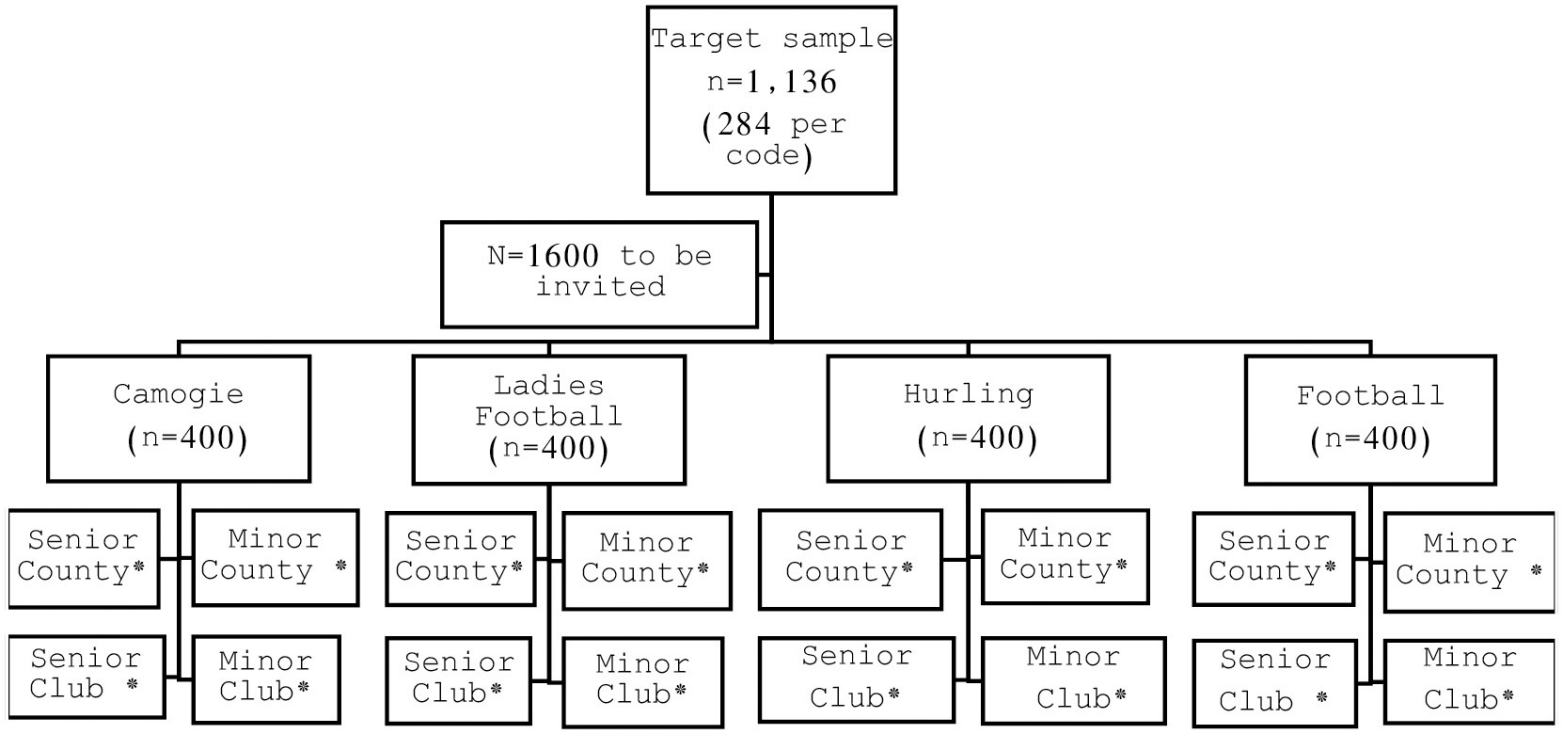
Recruitment process. *5 teams (approximately 20 players per team) were randomly selected from each sub-category to achieve a sample of 400 per code.

The sample in this study was selected to capture the diverse population of players who play multiple levels, grades and codes, representing most registered players in the sport. The Gaelic Games population was stratified into four codes (hurling, football, camogie and ladies football) and club and county teams, from minor (16–18 years) to adult age grades were identified in each province. Five teams (a minimum of 20 player per team) were then randomly selected from each subcategory ensuring an even distribution across all codes, age grades and levels (Fig 1).A minimum sample size of 284 players from each code was required to complete the survey based on a sample size calculation where FAIS prevalence was set at 0.8% (based on FAIS prevalence in other sports) [19–21], a confidence level of 95% and a precision of 0.01 for a finite population of 4000 senior intercountry players. It was intended that 400 players in each code would be invited to allow a contingency for non-response.

To be eligible for study inclusion, players were required to have played Gaelic games for their club and/or county for at least the previous year, participating in training/games a minimum of 2 times per week. Players were excluded if they did not provide written consent, or parental consent (if under 18 years). Players under the age of 16 and over 40 years were excluded from the study.

### Data collection

Self- reporting via survey was the most suitable mode of data collection given that many club teams may not have an appointed or consistent medical professional associated with the team. The managers of selected teams were contacted, informed about the study and permission to speak to the team was requested. Once permission was granted, the principal investigator (SJ) and research assistants attended a training session to inform potential participants (including players and parents in the case of U18’s) about the study, invite them to participate and distribute participant information leaflets and participant/parental consent forms. One week later, the researcher returned, and surveys were completed by players who returned signed consent forms. In the first two weeks of the Covid-19 pandemic lockdown in March 2020, surveys were also distributed online via Survey Monkey [23]. All documentation was circulated by the team’s manager on player/parent WhatsApp groups. A link for the survey was then circulated to players who consented to participate in the research.

The survey was developed through an iterative process with four musculoskeletal physiotherapists and academics and included a consensus meeting and three review rounds to determine the structure, focus and content of the survey (content validity). A draft survey was piloted with 30 GAA players which included 16 females and 14 males aged 16–32 years to gather feedback on understanding/interpretability and clarity of questions, layout and completion time (Face validity). The survey (S1 File) was refined until a final 20-item survey was developed, consisting of dichotomous, nominal, and open field responses with three main sections:

Section 1: Demographic information related to age, sex, grade, code and level of play, training and game time in both Gaelic games and additional sport/physical activity.
Section 2: Questions pertaining to hip and groin pain experienced within the last year (annual prevalence) and/or currently (point prevalence) including details on participation impact, symptom duration and requirement for medical attention.
Section 3. Questions pertaining to FAIS diagnoses within the last year (annual prevalence) including details on diagnostic criteria and medical professionals attended. This included details of medical professionals attended and 4 strict diagnostic criteria, which permitted classification of the presence of FAIS.

#### Definitions

The definition for hip and groin pain was informed by previous studies [24, 25] and was considered ‘*symptoms (including pain*, *stiffness*, *clicking*, *catching etc) located to the hip joint or surrounding soft tissues or at the junction between the anteromedial part of the thigh*, *including the proximal part of the adductor muscle bellies*, *and the lower abdomen which have impacted performance and/or caused players to reduce or stop participation in sport and/or receive treatment from a health professional’*. This was presented in plain language in the survey with symptom location illustrated on a body chart.

Femoroacetabular Impingement Syndrome (FAIS) was defined as a triad of symptoms, clinical signs and imaging findings as per the Warwick agreement and was presented in plain language in the survey [18]. The validation of FAIS diagnosis was based on players reporting the following four criteria:

1. Hip symptoms including pain/stiffness/clicking/catching/other.
2. Reproduction of pain with physical tests performed on the hip by a medical professional.
3. Confirmation of FAIS via X-Ray or MRI.
4. Diagnosed with FAIS by an orthopaedic consultant.

#### Indicators of severity

Respondents were asked to detail if their hip and groin pain stopped them participating in Gaelic games, selecting one of the following options:

*Yes = unable to participate in training and games for a consistent period of time*
*No = continued participation in training and games uninterrupted*
*Sometimes = reduced/intermittent participation in training and games*

Symptom duration was measured by the length of time respondents experienced their pain with categories ranging from less than 3 weeks to greater than 12 weeks, with an additional option for recurrent episodes or other durations of symptoms.

Respondents were asked to specify (yes/no) if they attended a medical professional with their hip and groin pain and if they received a diagnosis/explanation for their pain.

### Statistical analysis

Data were transferred from Excel to SPSS (2020, version 27.0; IBM Corp, Armonk, NY) for analysis. Participant demographic information was summarised in text as frequencies and means.

#### Point and annual prevalence of hip and groin pain and FAIS

Point prevalence and annual prevalence with 95% CI were calculated for all players as well as predefined subgroups for sex, code, level, and age, by dividing the number of people with hip and groin pain and FAIS by the total sample. Age was categorised according to Gaelic games age grades. Crude odds ratios with 95% CIs were calculated for sex, code, level, and age.

#### Predictors hip and groin pain

Logistic regression was used to explore factors/variables that are predictive of hip and groin pain within the last year. Preliminary analysis using chi squared tests and Mann Whitney tests examined relationships between hip and groin pain within the last year (yes/no) and individual predictors; sex (male/female team), participation in sport other than Gaelic Games (yes/no), code (Hurling/camogie and/or football and /or both), level of Gaelic games (club and/or school/college and/or county), grade (minor and/or U21 and/or senior), Gaelic games training (minutes/week), Gaelic games match (mins/week), training in other sport (mins/ week), match time in other sport (mins/week) and age (years). Variables with p-values <0.1 were considered for inclusion in the multivariable logistic model. Results of logistic regression analysis were expressed as odds ratios and 95% CIs. P values <0.05 were considered statistically significant.

Severity. Participation impact, symptom duration and requirement for medical attention were indicators of severity of hip and groin pain and descriptives are presented as percentages and frequency for all players and subdivided by sex. A Chi-squared test with post hoc analysis examined the association between sex, symptom duration and participation impact. Logistic regression explored if sex, symptom duration and participation impact were predictive of requirement for medical attention.

## Results

The covid 19 pandemic interrupted full recruitment and data collection but 1200 of the 1600 target population of Gaelic Games players from each of the four provinces in Ireland were invited to complete the survey. Eight hundred players responded to the survey, indicating a 67% response rate. Twenty-five surveys were deemed incomplete and excluded. Seven hundred and seventy-five (mean age 22.18±5.2; n = 438 males; n = 337 females) responses were therefore considered for analysis. Responses were collected via paper format (n = 479) and via an online platform, SurveyMonkey (n = 296). Players played multiple levels, codes and age grades and the distribution of the sample across these sub-categories is provided in S1 Table.

### Annual and point prevalence of hip and groin pain and FAIS

Over half (54.8%) of Gaelic games players experienced hip and groin pain within the last year, with a 22.8% point prevalence. Although fourteen respondents reported receiving a diagnosis of FAIS, within the last year, only eight met the four criteria required to be considered a FAIS case (Annual prevalence = 8/775; Proportion of hip and groin injuries = 8/425). Annual and point prevalence of hip and groin pain and FAIS across subgroups are presented in Table 1. A greater prevalence of hip and groin pain was observed in males across all subcategories and crude odds ratios indicate males are 1.5 times more likely to experience hip and groin pain and 1.3 times more likely to have FAIS than females. Furthermore, a greater proportion of hurlers/camogie players experience hip and groin pain compared to football players. A marginally greater proportion of players over 21 reported hip and groin pain compared to the under 21 category.

**Table 1:** The annual and point prevalence of hip and groin pain and annual prevalence of FAIS including subgroup prevalence rates and crude odds ratios for sex, code, level, and age.

	Hip and groin pain annual prevalence Percentage [95% CI] frequency (n)	Hip and groin pain point prevalence Percentage [95% CI] frequency (n)	FAIS Annual Prevalence Percentage [95% CI] frequency (n)
**Total (n = 775)**	54.8 [51.3, 58.3] (n = 425)	22.8 [19.8, 25.8] (n = 177)	1 [0.3, 1.7] (n = 8)
Females (n = 337)	49.3 [43.9, 54.7] (n = 166)	18.7 [14.5, 22.9] (n = 63)	0.9 [0, 1.9] (n = 3)
Males (n = 438)	59.1 [54.5, 63.7] (n = 259)	26 [21.9, 30.1] (n = 114)	1.1 [0.2, 2.1] (n = 5)
OR (Male/Female)	1.49 [1.12, 1.98]		1.3 [0.3, 5.4]
**Code (Female)**			
Camogie (n = 132)	51.5 (68)	15.9 (21)	2 (1.5)
Ladies football (n = 102)	41.2 (42)	17.6 (18)	1 (1)
Both (n = 103)	54.4 (56)	23.3(24)	0
OR (Camogie/Football)	1.52 [0.9, 2.56]		1.57 [0.14, 17.55]
**Code (Male)**			
Hurling(n = 134)	59 (79)	26.9 (36)	1.5 (2)
Football(n = 87)	54 (47)	21.8 (19)	1.1 (1)
Both (n = 217)	61.3 (133)	27.2(59)	0.9 (2)
OR (Hurling/Football)	1.22 [0.7, 2.1]		1.3 [0.1, 14.6]
**Level (Female)**			
Club only (n = 117)	46.2 (54)	16.2 (19)	0.9 (1)
Club and school/college (n = 53))	47.2 (25)	13.2 (7)	1.9 (1)
Club and county (n = 77)	54.5 (42)	29.9 (23)	1.3 (1)
Club, school/college and county(n = 90)	50 (45)	15.6 (14)	0
OR (Multiple level/Club only)	1.2 [0.77, 1.9]		1.1 [0.1, 11.86]
**Level (Male)**			
Club only (n = 194)	59.8 (116)	25.3 (49)	1 (2)
Club and school/college(n = 52)	57.7 (30)	28.8 (15)	1.9 (1)
Club and county (n = 91)	63.7 (58)	27.5 (25)	2.2 (2)
Club, school/college and county (n = 101)	54.5 (55)	24.8 (25)	0
OR (Multiple level/Club only)	0.95 [0.65, 1.4]		1.2 [0.2, 7.22]
**Age (Female)**			
Under-18 (n = 109)	44 (48)	11.9 (13)	0.9 (1)
19-21(n = 94)	48.9 (46)	21.3 (20)	1.1 (1)
Over 21(n = 129)	54.3 (70)	23.3 (30)	0.8 (1)
OR (Over 21/Under 21)	1.4 [0.9, 2.1]		0.8 [0.1, 8.8]
**Age (Male)**			
Under 18 (n = 94)	59.6 (56)	23.4 (22)	0
19-21(n = 112)	56.3 (63)	21.4 (24)	0
Over 21(n = 229)	60.7 (139)	29.3 (67)	2.2 (5)
OR (Over 21/Under 21)	1.1 [0.8, 1.7]		-

OR: Crude Odds Ratios

### Predictors of hip and groin pain

Univariable analysis indicated sex (ChiSq = 7.49, p = 0.006), participation in additional sport/physical activity (ChiSq = 13.69, p<0.001), code (ChiSq = 6.91, p = 0.032), and age (Z = -1.89, p = 0.05) were significantly associated with the presence of hip and groin pain within the last year. Level and grade were not associated with hip and groin pain. Overall training and match time in Gaelic games and additional sport was marginally higher in players with hip and groin pain but this was not statistically significant (Table 2).

**Table 2:** Means and 95% confidence intervals for training and game time (minutes/week) in Gaelic games and additional sport/physical activity.

	Hip and groin pain within the last year		Total
	No	Yes	
	(n = 350)	(n = 425; m = 259, f = 179)	
	Mean [95% CI]	Mean [95% CI]	
	Minutes/week	Minutes/week	
**Gaelic Games**			
**Training time (n = 775)**	276.3 [260.8, 291.9]	290.1 [276.7, 303.6]	283.9 [273.7, 294.1]
**Males (n = 438)**	284.22 [260.5, 307.9]	283.6 [267, 300.3]	283.9 [270.1, 297.6]
**Females (n = 337)**	268 [248, 288]	300.3 [277.5, 323.2]	284 [268.8, 299.1]
**Match time**	108 [101.9, 114.1]	110.8 [104.9, 116.8]	109.5 [105.3, 113.8]
**Male (n = 438)**	105.4 [96.4, 114.4]	104.3 [97, 111.7]	104.7 [99.1, 110.4]
**Female (n = 337)**	110.7 [102.5, 119]	121 [111.1, 130.8]	115.8 [109.4, 122.2]
**Additional Sport**	**No (n = 253)**	**Yes (n = 344; m = 209, f = 135)**	**Total**
**Training time (n = 597)** ^+^	150.59 [133.7, 176.4]	159.27 [146.1, 172.5]	155. [145.2, 166]
**Males (n = 340)**	163.2 [133.2, 193.2]	150.6 [139.7, 161.4]	155.4 [142.2, 168.7]
**Females (n = 257)**	137.1 [123.1, 151]	172.8 [143.4, 202.1]	155.8 [139, 172.6]
**Match Time (n = 604)** ^++^	20.7 [14.9, 26.4]	22.4 [18.1, 26.7]	21.52 [18, 25]
**Male (n = 347)**	23.9 [14.5, 32.3]	22.6 [17, 28.1]	23.1 [18.1, 27.9]
**Female (n = 257)**	17.5 [10.9, 24.2]	21.2 [14.2, 28.3]	19.5 [14.7, 24.3]

SD: Standard Deviation; n = number; m = male; f = female;

^**+**^ = 26 missing values (18 = yes, 8 = no; n = 20 males, n = 6 females;)

^**++**^ = 19 missing values (13 = yes, 6 = no; n = 6 females; n = 13 males

The multivariable logistic regression therefore included sex, participation in other sport, code and age. The regression model was statistically significant (x(5) = 26.983, p<0.05) and correctly classified 58.1% of cases. Males were 1.4 (95% CI 1, 1.8) times more likely to report hip and groin pain throughout the year than females (p = 0.043). Gaelic Games players who participate in additional sports are 1.95 (95% CI 1.35, 2.79) more likely to experience hip and groin pain (p<0.001). Players who play both hurling/camogie and football are 1.5 (95% CI 1, 2.2) times more likely to report hip and groin pain throughout the year than those who play football only (p = 0.032) A total of 59.1% of players playing both hurling/camogie and football reported hip and groin pain, while 47.1% of football players reported hip and groin pain within the last year. Age was not a significant predictor (p>0.05).

### Severity

#### Participation impact

Almost half (48.8%) of players continued to play sport with their hip and groin pain, while 18.7% stopped participating in sport and approximately one third (32.5%) experienced reduced participation. Similar patterns were observed for males and females regarding participation impact (Table 3), with no significant sex difference (ChiSq = 1.68, p>0.05).

**Table 3:** Symptom duration and participation impact for male and female players who reported hip and groin pain within the last year.

	0–3 weeks % (n)	4–8 weeks % (n)	9–12 weeks % (n)	More than 12 weeks % (n)	Recurrent episodes % (n)	Other % (n)	Total
**Total (n = 415)** ^+^	40 (166)	16.4 (68)	2.4 (10)	4.8 (20)	33.5 (139)	2.9 (12)	
**Continued to play**	63.3 (105) *	23.5 (16) *	10 (1)	25 (5)	49.6 (69)	50 (6)	48.8 (206)
**Unable to play**	13.9 (23)	29.4 (20)	30 (3)	35(7)	17.3 (24)	16.7 (2)	18.7 (79)
**Reduced play**	22.9 (38)	47.1 (32)	60 (6)	40 (8)	33.1 (46)	33.3 (4)	32.5 (137)
**Males (n = 255)**	40.6 (104)	14.8 (38)	2.3 (6)	6.3(16)	32.8 (84)	3.1 (8)	
**Continued to play**	58.7 (61) *	23.7 (9) *	0 (0)	25 (4)	50 (42)	50 (4)	46.8 (121)
**Unable to play**	17.3 (18)	31.6 (12)	16.7 (1)	37.5 (6)	17.9 (15)	12.5 (1)	20.5 (53)
**Reduced play**	24 (25)	44.7 (17)	83.3 (5)	37.5 (6)	32.1 (27)	37.5 (3)	32.6 (84)
**Females (n = 160)** s	39 (62)	18.9 (30)	2.5 (4)	2.5 (4)	34.6 (55)	2.5 (4)	
**Continued to play**	71 (44) *	23.3 (7) *	25 (1)	25 (1)	49.1 (27)	50 (2)	51.8(85)
**Unable to play**	8.1 (5)	26.7 (8)	50 (2)	25 (1)	16.4 (9)	25 (1)	15.9(26)
**Reduced play**	21 (13)	50 (15)	25 (1)	50 (2)	34.5 (19)	25 (1)	32.3(53)

N = number;

^**+**^ = 10 missing values; A post hoc analysis was carried out using chisquared residuals. A z score >1.96 is considered statistically significant but to reduce the risk of type 1 error, an adjusted p value was used (0.05/18 cells = 0.003).

* = statistically significant.

#### Symptom duration

The majority of hip and groin pain episodes had short symptom duration (40%), with a high recurrence rate (33.5%). Symptom duration for males and females is presented in Table 3. Similar patterns were also observed in symptom duration between males and females and there was no significant sex difference in symptom duration (ChiSq = 12.62, p>0.05). However, Chi squared test and post hoc analysis found a significant association between symptom duration less than 3 weeks and continued participation in sport and symptom duration of 4 to 8 weeks and continued participation (Table 3).

#### Requirement for medical attention

Overall, 60% (n = 256) of players sought medical attention for their hip and groin pain. Players who experienced reduced participation in sport were 3.3 times more likely to seek medical attention than those who continued to play (p<0.001). Players who stopped participation were 3.9 times more likely to seek medical attention that those with reduced participation (p = 0.002). Males were 1.6 times more likely than females to seek medical attention with their hip/groin pain (p = 0.042). Symptom duration was not predictive of requirement for medical attention (p>0.05). A total of 60% (n = 154) of those that attended a medical professional indicated that they had been given a diagnosis. There was a wide range of diagnoses given for hip and groin pain with 43 different categories, grouped based on similar terminology and anatomical location (S2 Table).

## Discussion

This is the first epidemiological study on hip and groin pain in a national representative sample of Gaelic Games players inclusive of all codes from minor grade to adult. A high annual prevalence of hip and groin pain (54.8%) was reported with FAIS representing a relatively small number of complaints. Male sex and greater exposure to Gaelic games and other sport/physical activity are significant factors in predicting players who may experience hip and groin complaints. It is imperative to consider the severity of hip and groin issues in terms of participation impact, symptom duration and requirement for medical attention to give context to the extent of problem and subsequently better inform management and preventative practices.

Over half of players reported an episode of hip and groin pain (54.8%) but almost half of these players continued to play sport and had a short symptom duration with the majority resolving withing 3 weeks. Haroy et al (2017) reported similar results in Norwegian soccer players where, over a 6 week period in the competitive season using the OSTRC overuse injury questionnaire, 59% (n = 112) of males and 45% (n = 20) of females, compared to 59% and 49% in this study, reported one episode of a groin complaint but only 34% (n = 38) males and 20% (n = 4) females were time loss injuries [12]. A study investigating the prevalence of hip and/or groin pain in sub-elite male football players reported 49% of 695 players experienced pain during the last season but similar to our findings, just over half (54%) of respondents stated no games were missed, and 40% of these reported symptoms lasting 1 to 2 weeks [13]. Collectively, these studies support the trend that time-loss injury surveillance methods may only capture one fifth to one third of hip and groin complaints.

The prevalence rates of hip and groin pain resulting in complete cessation of participation in sport (18.7%) is consistent with previous research in men’s and women’s football with groin pain accounting for up to 19% of all time loss injuries [4, 25, 26]. Previous Gaelic games studies documented hip and groin related pain separately but when combined, time loss rates are comparable or slightly lower than in this study. In a hurling injury surveillance study, 10.3% of all injuries were attributed to the groin/pelvis region over a 4-year surveillance period, of which 2.3% of injuries were specific to the hip [7]. Similar prospective injury surveillance studies on elite male Gaelic football players reported 9.4–14.4% of all lower limb injuries were to the pelvis/groin, with the hip accounting for 3.1–3.4% [9, 10]. The only study to report hip and groin injury in the female codes was based on collegiate ladies football, where 2.5% injuries were attributed to the hip and 2.5% to the groin [11]. These rates are considerably lower than time loss rates for females in this study but did not include camogie players, represented collegiate players only from a single institute and reported proportion of total injuries rather than prevalence.

It is well documented that despite the presence of symptoms and pain from groin injuries, many athletes continue to train and compete, in some cases with impaired performance [4, 12]. While previous studies record either time-loss and/or non-time-loss complaints, this current study had the additional category to account for players who experienced reduced participation. This represents a group who are likely to have reduced performance and/or participation with intermittent time-loss during an episode of pain and account for almost one third of players with hip and groin pain. This cohort, who intermittently play with pain and with potentially longer duration or recurrent symptoms, are almost 4 times less likely to seek medical attention than those with time-loss injury and therefore may be more vulnerable to chronicity.

Male athletes are consistently reported to be at an increased risk of sustaining groin problems compared to females, a trend observed in this study where male players were 1.5 times more likely to experience hip and groin pain. These ratios are slightly lower than previous studies with odds ratios of 3.1 and risk ratios of 1.4–2.5 in elite male team sport, predominantly soccer, compared to female counterparts [4, 5, 12, 19]. Players who participate in additional sport/physical activity and play both codes of Gaelic games are more likely to experience hip and groin pain, suggesting that engagement in multiple different sports/physical activity increases the risk of suffering hip and groin pain. While training load variables have been cited as risk factors for hip and groin injury, a history of previous injury, weak adductors and abductors and older age are the predominant risk factors for hip and groin pain [16]. Furthermore, a lower level of sport-specific training has been associated with increased risk of groin injuries which may occur when playing multiple sports [17].

This is the first study to provide prevalence rates for FAIS in Gaelic games. Of the fourteen reports of FAIS, only eight satisfied the diagnostic criteria, with males being 1.3 times more likely to be diagnosed with FAIS. In previous epidemiological studies, FAIS is consistently reported as a proportion of hip and groin injury rather than total injuries, and rates are marginally lower in previous studies on team sports than in this study (8/425). Data from the NCAA from 2009–2014 indicated that FAIS was present in 1.4% of cases of hip and groin pain across a multitude of sports representing both sexes [19]. Specifically, in women’s soccer, NCAA data indicated hip impingement was reported in 0.9% of hip and groin injuries [21]. Interestingly, two separate studies on males indicated FAIS as a cause of 0.3%-0.7% of hip and groin injuries, a slightly lower rate than reported in female soccer and this current study [20, 24]. Two studies reported much higher rates where FAIS represented 3% of the total population and 13.6–13.9% of hip injury in men’s football and NCAA athletes, with no sex difference observed in NCAA sports [26, 27].Although, male athletes are more likely to have mixed (cam and pincer) morphology, and pincer morphology is more common in females, there is no agreement on prevalence rates of FAIS across sexes [28].

Some limitations should be noted in this study. Although self-reporting can lead to recall bias and overestimation of prevalence, measures were taken to reduce the impact of this. Players were only asked about pain in one body region within the last year to reduce recall bias; a body chart accompanied by a plain language definition of hip and groin pain ensured clarity of what hip and groin meant; collection of information on severity provided important injury context; detailed questioning on symptoms, clinical and imaging findings consistent with the Warwick agreement reduced the potential for unsubstantiated claims of FAIS diagnosis. Furthermore, the IOC advocates self-reporting in such instances where there may not be access to suitable medical professionals to report injuries or where the problem may not limit athletic participation. The cross-sectional nature of this study meant no cause-effect relationship could be ascertained but the study design facilitated reaching a large sample of Gaelic games players that are underrepresented in research.

These findings may be applicable to other field invasion team sports, particularly amateur and female cohorts for which hip and groin injury data is lacking. This study provides medical professionals with a snapshot of the landscape of hip and groin pain in Gaelic games highlighting the dynamic nature of these complaints. Prospective epidemiological studies are needed to monitor the long-term outcome of players who experience complete, reduced and indeed continued participation during an episode of hip and groin pain. The provision of this epidemiological data ultimately represents a crucial step for the development of strategies to reduce and prevent the occurrence of hip and groin problems.

## Conclusion

Hip and groin pain is a frequent complaint in male and female Gaelic games players and a small proportion of these players will be diagnosed with FAIS. Almost half of Gaelic games players continue to participate in sport with hip and groin pain and this cohort experience short duration of symptoms. Concerningly, one fifth of players will cease participation in sport due to their hip and groin pain while one third will experience reduced participation. Males, players who play both codes of Gaelic games and partake in additional sport/physical activity are more likely to experience hip and groin pain and should be carefully monitored to prevent development of symptoms. Future injury surveillance and epidemiological studies should record all hip and groin complaints irrespective of time-loss to identify players who may struggle to return to full participation or preinjury performance levels in sport.

## Supporting information

S1 File Player survey. (PDF)

S1 Table Frequency of age grade, code and level of Gaelic games for all respondents. (PDF)

S2 Table Frequency of diagnoses for hip and groin pain. Diagnoses are categorised according to similar terminology and anatomical locations.
(PDF)

S1 Dataset (XLSX)
