# Supplementary material for: The epidemiology of hip and groin pain and Femoroacetabular Impingement Syndrome (FAIS) in male and female Gaelic games players
Source: PLoS One. 2024 Sep 25;19(9):e0309027. doi: 10.1371/journal.pone.0309027 (PMC11423975; doi:10.1371/journal.pone.0309027)
Supplement: S1 File — (PDF) [file pone.0309027.s001.pdf]

GAA Player Questionnaire

The purpose of this questionnaire is to gather information about hip and groin pain in GAA players.

Please complete all questions in Section 1 below.

Section 1: Personal information

Q1. With which Gaelic Games team do you play?

Male team ☐ Female team ☐

Q2. Age _______________

Q3. In the table below, please tick the level(s) and category(ies) of Gaelic games that you have participated in over the last year. You may select more than one option if applicable.

	County					School/ College	Club				
	Senior	Inter-mediate	Junior	U21	Minor (u17's)	------------ ---------	Senior	Inter-mediate	Junior	U21	Minor (u17's)
Hurling											
Football											
Camogie											
Ladies Football											

Q4. In what position do you normally play? Please tick appropriate box(es).

	County		School/College		Club	
	Hurling/ Camogie	Football	Hurling/ Camogie	Football	Hurling/ camogie	Football
Forward/Offense						
Midfield						
Back/defender						
Goalkeeper						

Q5. Over the last year, on average, how many **minutes per week** did you play Gaelic Games/GAA?

	Training (minutes per week)	Games (minutes per week)
Club		
School/College		
County		

Q6. (i) Do you or have you participated in any other sport or physical activity (e.g. gym) over the last year? Yes ☐ No ☐

(ii)If yes, please specify, in the table below, any other sport(s)/physical activity that you partake in and the number of **minutes per week** that you participate in this sport/activity.

Sport or Physical Activity	Training (minutes per week)	Games (minutes per week)

In section 2 below, you will be asked about any hip or groin pain and/or symptoms that you currently experience or have experienced within the past year.

Note: Hip and/or groin pain is described as symptoms (including pain, stiffness, clicking, catching, weakness etc) in the areas marked on the body chart below, which have impacted performance, caused you to reduce or stop participation in your sport and/or receive treatment from a health professional.)

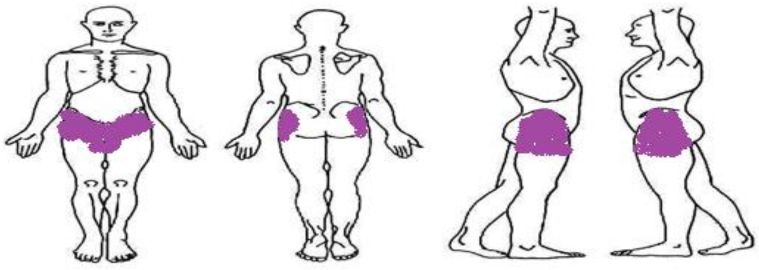

Section 2: Hip/Groin pain

Q1. (i)Have you experienced hip/groin pain over the last year?

Yes ☐ No ☐

(ii)If yes, for how long did you experience this pain?

3 weeks or less ☐ 4 to 8 weeks ☐ 9 to 12 weeks ☐

More than 12 weeks ☐ Recurrent episodes of pain ☐

Other (please specify)________________________________

Q2. Do you currently experience this hip/groin pain?

Yes ☐ No ☐

Q3. Does/did your hip/groin pain stop you from participating in sport?

Yes ☐ No ☐ Sometimes ☐

Yes= unable to participate in training and games for a consistent period of time

No= continued participation in training and games uninterrupted

Sometimes=reduced/intermittent participation in training and games

<u>If you answered NO to ALL questions above (Section 2: Question 1, 2 and 3), you do not need to answer any further questions. Thank you for taking the time to complete this questionnaire.</u>

<u>If you answered YES to Question 1 and/or Question 2 above, please answer the remaining questions in Section 2 below.</u>

Q4. What is/was the location of your hip/groin pain? Please mark on chart below

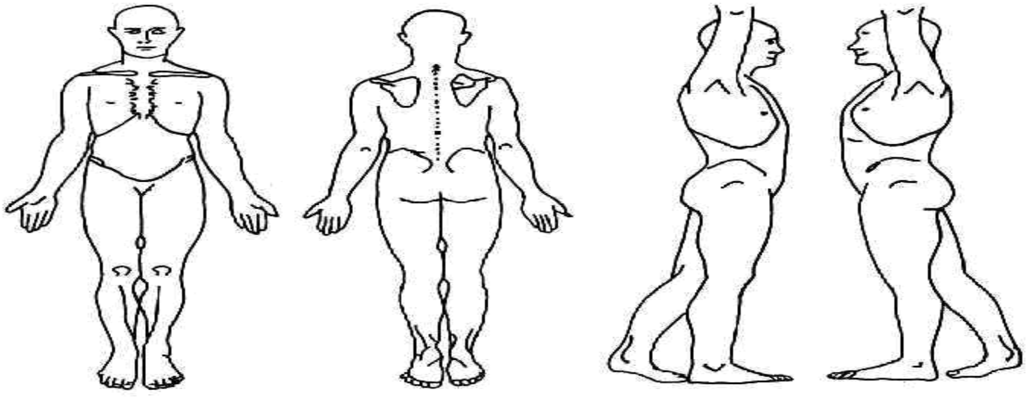

Q5. Is/was your hip or groin pain in your dominant or non-dominant leg (note your dominant leg is your kicking leg)?

Dominant ☐ Non-dominant ☐ Both ☐

Q6. (i) Have you attended a health professional with your hip/groin pain?

Yes ☐ No ☐

(ii) If yes, which of the following health professionals did you attend? (You may select more than one option if applicable.)

Team Doctor ☐ Family Doctor ☐ Physiotherapist ☐ Sport Physician ☐

Consultant Orthopaedic Surgeon ☐ Rheumatologist ☐ Athletic Therapist ☐

Sports Rehabilitator ☐ Physical Therapist ☐ Not sure ☐

Other (please specify)________________________________

(iii) Did the medical professional that you attended tell you if you had a particular injury or problem with your hip/groin?

Yes ☐ No ☐

If yes, what injury/problem were you told you had?

____

____

In Section 3 below, you will be asked about your experiences of Femoroacetabular Impingement syndrome (FAIS) also known as FAI or hip impingement. FAI/hip impingement occurs when there is abnormal contact between the ball and socket of the hip joint resulting in symptoms such as pain, stiffness and clicking.

If you have NOT been diagnosed with FAI/hip impingement, you do not need to answer the questions in Section 3. Thank you for taking the time to complete the questionnaire.

If you HAVE been diagnosed with FAI/hip impingement, please answer the questions in section 3 below.

****

Section 3: Femoroacetabular Impingement Syndrome (also known as FAI/hip impingement)

Q1. Have you ever been diagnosed by a health professional with hip impingement?

Yes ☐ No ☐

Q2. Have you been diagnosed with hip impingement by a health professional within the last year?

Yes ☐ No ☐

Q3. What health professional diagnosed you with hip impingement?

Team Doctor ☐ Family Doctor ☐ Physiotherapist ☐ Sport Physician ☐

Consultant Orthopaedic Surgeon ☐ Rheumatologist ☐ Athletic Therapist ☐

Sports Rehabilitator ☐ Physical Therapist ☐ Not sure ☐

Other (please specify) ________________________________

Q4. How long did it take for your hip impingement to be diagnosed from the first time you experienced pain?

less than 1 month ☐ 1-3 months ☐ 4-6 months ☐ 7-9 months ☐

10-12 months ☐ Other (please specify) ____

Q5. Have you experienced any of the following symptoms? You may select more than one option, if applicable.

Pain ☐ Stiffness ☐ Catching/clicking in the hip ☐ None ☐

Other (please specify) ____

Q6. Did the health professional who diagnosed you with hip impingement perform a physical exam/tests on your hip? (physical exam and tests may involve moving your hip in different directions to try reproduce your pain, measuring range of motion in your hip, testing muscle strength around the hip)

Yes ☐ No ☐

Q7. Did you have any imaging procedures carried out on your hip to confirm hip impingement? You may select more than one option if applicable.

X-ray ☐

MRI * ☐

MRA** ☐

CT*** ☐

None of the above ☐

*Magnetic resonance imaging (MRI) is a technique that uses a magnetic field and radio waves to create detailed images of the organs and tissues within your body.

**Magnetic Resonance Arthrography (MRA) IS similar to MRI but a dye is injected into the hip joint

***computerized tomography (CT) scan combines a series of X-ray images taken from different angles around your body and uses computer processing to create cross-sectional images (slices) of the bones, blood vessels and soft tissues inside your body. CT scan images provide more-detailed information than regular X-rays do.

Q8. Have you received any treatment for your hip impingement?

Yes ☐ No ☐

If yes, what treatment have you received? (you may select more than one option, if applicable)

Physiotherapy ☐ Exercise therapy ☐ Surgery ☐ Injections ☐ Oral Medication ☐

Advice ☐ No treatment ☐ Other ____________________ _________________

Thank you for taking the time to complete this questionnaire.

<u>If you have been diagnosed with Femoroacetabular impingement Syndrome (FAI/hip impingement) please read below</u>

There will be a follow-on study taking place in which the researcher will be interviewing players who have been diagnosed with hip impingement. The aim of this interview is to explore your experiences of hip impingement and its impact on your sporting and daily life. This information would be valuable in giving us a deeper understanding of the condition and guiding future research and care of GAA players with hip impingement.

If you would like further information on this or would like to volunteer to take part in this study, please provide your contact details below or feel free to come talk to me today. Your name and contact details will be securely stored and will not be shared or accessed by anyone other than the researcher.

Name (please print): ____

Team: ____

Phone number: ____________________________________
