# Supplementary material for: The epidemiology of hip and groin pain and Femoroacetabular Impingement Syndrome (FAIS) in male and female Gaelic games players
Source: PLoS One. 2024 Sep 25;19(9):e0309027. doi: 10.1371/journal.pone.0309027 (PMC11423975; doi:10.1371/journal.pone.0309027)
Supplement: S1 Table — (PDF) [file pone.0309027.s002.pdf]

S2 Table. Frequency of Age grade, Code and Level of Gaelic Games for all respondents

Age Grade	Code	Levels				Total
		club only	club and school	club and county	club school and county	
**Minor**	**Hurling/Camogie**	7	10	14	24	55
	**Football**	10	16	13	17	56
	**Both**	4	15	4	69	92
	**Total**	21	41	31	110	203
**Under 21**	**Hurling/Camogie**	20	17	13	29	79
	**Football**	16	8	14	19	57
	**Both**	20	22	12	16	70
	**Total**	56	47	39	64	206
**Adult**	**Hurling/Camogie**	78	4	41	6	129
	**Football**	36	3	29	4	72
	**Both**	116	10	26	5	157
	**Total**	230	17	96	15	358
**Total**	**Hurling/Camogie**	105	31	68	59	263
	**Football**	62	27	56	40	185
	**Both**	140	47	42	90	319
	**Total**	307	105	166	189	767

N=3 missing values for age for males; n-5 missing values for age for females
