# Supplementary material for: The epidemiology of hip and groin pain and Femoroacetabular Impingement Syndrome (FAIS) in male and female Gaelic games players
Source: PLoS One. 2024 Sep 25;19(9):e0309027. doi: 10.1371/journal.pone.0309027 (PMC11423975; doi:10.1371/journal.pone.0309027)
Supplement: S2 Table — Diagnoses are categorised according to similar terminology and anatomical locations. (PDF) [file pone.0309027.s003.pdf]

S3 Table. Frequency of diagnoses for hip and groin pain. Diagnoses are categorised according to similar terminology and anatomical locations

	Frequency	Percent
hip flexor strain/tight/weak/pulled/tear/hyperextension of hip flexor/muscle at front of hip/inflamed	21	2.7
groin strain/tear/graded/groin muscle pulled away from tendon/groin tendonitis	15	1.9
hip impingement/cam/fai/excessive bone formation/bone deformities/FAIS/femoroacetabular impingement	15	1.9
inflammation of hip adductor/add tear/add strain/add tendonopathy	10	1.3
inactive/weak/tight glutes/imbalance	8	1.0
strain/tear/tight	7	.9
muscle tightness/imbalance/dominant on other side	7	.9
symphysis pubis/osteitis pubis	5	.6
tight it band/it band related	3	.4
tight hips+/- causing groin and testicle pain	2	.3
tfl muscle injury	2	.3
trapped sciatic nerve/nerve impingement in hip-sciatica	2	.3
pelvic wasn't aligned/malalignment of pelvis	2	.3
poor movement in hips/restricted movementt hips/ stiff hips	2	.3
anterior pelvic tilt/tilted pelvis	2	.3
hip pain/stiffness//stiffness due to previous knee injury	2	.3
gilmores groin	1	.1
hip joint inflammation	1	.1
hip ball slightly flattened	1	.1
tight abductor/abductor tear	1	.1
displaced sacroiliac joint	1	.1
weak lower back causing hip pain	1	.1
weak outer hip	1	.1
Hernia	1	.1
no definite injury	1	.1
Overuse	1	.1
hip moved	1	.1
tightness from hip impingement surgery in 17	1	.1
grade 2 prox ham tear	1	.1
quad dominant causing strained muscles in hip	1	.1
Gluteal tendonopathy/tendonitis	1	.1
l4/5 disc	1	.1
damaged tendon	1	.1
coxa saltans	1	.1
pulled muscle	1	.1
Hypermobility	1	.1
hips were out of line and my back muscles were too short	1	.1
clicking hips	1	.1
torn labrum	1	.1
cannot recall/not sure	9	1.2

**More than one diagnosis/pathology**		
hip impingement/cam/fai/excessive bone formation/bone deformities/FAIS/femoroacetabular impingement AND damaged/torn cartilage	2	.3
hip impingement/cam/fai/excessive bone formation/bone deformities/FAIS/femoroacetabular impingement AND capsule degeneration	1	.1
hip impingement/cam/fai/excessive bone formation/bone deformities/fais/femoroacetabular impingement AND chronic fatigue	1	.1
hip impingement/cam/fai/excessive bone formation/bone deformities/FAIS/femoroacetabular impingement AND torn labrum	2	.3
poor movement in hips/restricted movement hips/ stiff hips AND muscle tightness/imbalance/dominant on other side	1	.1
poor movement in hips/restricted movement hips/ stiff hips AND hip impingement/cam/fai/excessive bone formation/bone deformities/fais/femoroacetabular impingement	1	.1
symphysis pubis/osteitis pubis AND groin strain/tear/graded/groin ms pulled away from tendon/groin tendonitis	1	.1
symphysis pubis/osteitis pubis AND hip impingement/cam/fai/excessive bone formation/bone deformities/FAIS/femoroacetabular impingement	1	.1
inactive/weak/tight glutes/imbalance AND hip flexor strain/tight/weak/pulled/tear/hyperextension of hip flexor/ms at front of hip/inflamed	1	.1
inactive/weak/tight glutes/imbalance AND hip flexor strain/tight/weak/pulled/tear/hyperextension of hip flexor/ms at front of hip/inflamed	1	.1
groin strain/tear/graded/groin muscle pulled away from tendon/groin tendonitis AND tight abductor/abductor tear AND symphysis pubis/osteitis pubis	1	.1
groin strain/tear/graded/groin muscle pulled away from tendon/groin tendonitis AND gluteal tendonopathy/tendonitis	1	.1
groin strain/tear/graded/groin muscle pulled away from tendon/groin tendonitis AND symphysis pubis/osteitis pubis	1	.1
gilmores groin AND hip impingement/cam/fai/excessive bone formation/bone deformities/FAIS/femoroacetabular impingement	1	.1
